# Mortality in hemodialysis patients in Ethiopia: a retrospective follow-up study in three centers

**DOI:** 10.1186/s12882-022-03053-6

**Published:** 2023-01-04

**Authors:** Beza Zewdu Desta, Abel Fekadu Dadi, Behailu Tariku Derseh

**Affiliations:** 1 Ethiopian Food, Medicine Administration and Health Care Authority, Addis Ababa, Ethiopia; 2 Menzies School of Health Research, Charles Darwin University, Darwin, Australia; 3 Departments of Epidemiology and Biostatistics, College of Medicine and Health Sciences, University of Gondar, Gondar, Amhara Ethiopia; 4 School of Public Health, Asrat Woldeyes Health Sciences Campus, Debre Berhan University, Debre Berhan, Ethiopia

**Keywords:** Mortality, Incidence, Hemodialysis patients, Chronic kidney diseases

## Abstract

**Background:**

The prevalence of chronic kidney disease (CKD) is between 10 and 15% worldwide. Ethiopia is seeing a consistent increase in the number of dialysis patients. Patients on chronic hemodialysis have high mortality rates, but there is little information available in Ethiopia. Thus, this study looked into patient mortality and the factors that contributed to it at three dialysis centers in Addis Ababa for hemodialysis patients.

**Method:**

A facility-based retrospective follow-up study was employed among End-Stage Renal Disease patients on hemodialysis from 2016 to 2020 at St. Paul Millennium Medical College (SPMMC), Zewditu Memorial Hospital (ZMH), and Menelik II Hospital. The proportional hazard assumption was checked by using the Log (-log (St)) plots and tests. Life-table analysis was fitted to estimate the one and five-year’s survival probability of these patients and Cox Proportional regression analysis to model the predictors of mortality at *p*-value < 0.05.

**Result:**

Over the course of 2772 person-months, 139 patients were tracked. Of these patients, 88 (63.3%) were male and the mean age (± SD) of the patients was 36.8 (± 11.9) years. During the follow-up period, 24 (17%) of the patients died, 67 (48.2%) were alive, 43 (30.9%) received a kidney transplant, and 5 (3.6%) were lost to follow-up. The mean survival time was 46.2 months (95% CI: 41.8, 50.5). According to estimates, there were 104 deaths per 1000 person-years at the end of the follow-up period. The likelihood that these patients would survive for one and 5 years was 91%% and 65%, respectively. Our analysis showed that patients with hypertension (Adjusted Hazard Rate (AHR) = 4.33; 95% CI: 1.02, 34.56), cardiovascular disease (AHR = 4.69; 95% CI: 1.32, 16.80), and infection during dialysis (AHR = 3.89; 95% CI: 1.96, 13.80) were more likely to die.

**Conclusion:**

The hemodialysis patients' death rate in the chosen dialysis facilities was high. Preventing and treating comorbidities and complications during dialysis would probably reduce the mortality of CKD patients. Furthermore, the best way to avoid and manage chronic kidney disease is to take a complete and integrated approach to manage hypertension, diabetes, and obesity.

## Introduction

Chronic kidney disease (CKD) is a major public health problem that challenges health systems around the world [1]. Chronic kidney disease is a progressive loss of kidney function over a period of months or years. Kidney Disease Improving Global Outcomes (KDIGO) guidelines classified the severity of CKD into five stages. A clinically significant Stage 5 CKD or end-stage renal disease is the most deliberating stage in which the patient should get Renal Replacement Therapy(RRT) [2–4].

Although there is a limitation of data about the prevalence of CKD, few studies indicate that Renal disease has become a significant public health problem in Ethiopia. According to a cross-sectional study, the prevalence of CKD in Ethiopia is estimated to be 12.2% and has risen in recent years along with the rise in diabetes and hypertension cases. The prevalence of CKD is as high as 41.0% in ages < 35 years and 62% in males [5].

Dialysis is the first-line treatment for individuals with severe CKD due to the scarcity of organs for transplantation and the high risks associated with transplantation for the vast majority of patients around the world. Dialysis extends and improves the quality of life of kidney failure patients, and this necessitates an ongoing evaluation of the process in order to enhance treatment outcomes [2, 6].

Between 1990 and 2010, the number of deaths attributed to CKD approximately doubled globally, ranking as the 18th leading cause of mortality [7]. More than 70% of patients with end-stage renal illness are predicted to reside in low-income countries by 2030 [8].

Patients with end-stage renal disease (ESRD) are unable to sustain life without dialysis support [9]. About two to three times each week, dialysis would take three to four hours. The duration of each session depends on how well the patient's kidneys work, and how much fluid weight the patient has gained between treatments [9]. Arterio-Venous Fistula (AVF), Arterio Venous Graft (AVG), and Central Venous Catheter (CVC) are the three primary vascular accesses (VA) used in hemodialysis (HD) treatment in Ethiopia [10]. All types of VA have their own risks and expenses. The clinical practice guidelines recommend, AVF as the first choice because of its reduced associated complications, morbidity, and mortality compared with the AVG and CVC [11].

Hemodialysis is the only type of dialysis being offered to ESRD patients in Ethiopia as peritoneal dialysis is not available. The vast majority of ESRD patients receive palliative care because they cannot afford or access a dialysis facility. According to a survey completed in September 2021, there are 35 hemodialysis units in Ethiopia. The federal or local governments subsidize the cost of 11 units at government-run hospitals; the remaining units are privately owned for profit. The remaining four dialysis units are standalone, while the other thirty-one are housed in either a hospital or a clinic. A facility may have anywhere from 10 and 250 patients, with a median of 22 (IQR = 23). There are an estimated 1132 patients undergoing hemodialysis, which means that the prevalence is roughly ten per million people (10 ppm) [12].

The high rate of mortality among dialysis patients after initiation of the therapy is attributed to factors such as comorbidities, blood markers (albumin and hemoglobin), and type of vascular access [13]. Duration of dialysis per session, hypertension, and infection status significantly affect the survival rate of CKD patients on hemodialysis [14].

A study on the survival pattern of hemodialysis patients in Ethiopia shows that 45.1% of death occurs during dialysis treatment and 23.1% of the patient died within the first 90 days of initiation of dialysis, and only 42.1% of the patients survive more than a year. Septicemia (34.1%), cardiovascular disease (29.3%), and uses of the catheter as vascular access was associated with short and long-term survival of patients treated with hemodialysis [7]. There is limited data reporting mortality and its predictors among chronic hemodialysis kidney patients that we intended to investigate in this study. Thus, the overall goal of this study is threefold: first, to estimate the mortality rate of chronic hemodialysis patients in a chosen hospitals, second, to identify significant predictor variables of mortality in hemodialysis treatment Centers, and third, to compare the length of time that patients have survived after starting hemodialysis.

## Methodology

### Study setting and period

A retrospective follow-up study was conducted to analyze data of patients enrolled in maintenance hemodialysis in three hospitals in Addis Ababa (St. Paul Millennium Medical College (SPMMC), Zewditu Memorial Hospital (ZMH), and Menelik II) from ${1}^{st}$ January 2016 to 31^st^ December 2020. Addis Ababa has a population size of 3,435,028 of whom 1,809,577 are females [15]. There are 13 public and 34 private hospitals in the centers providing different public health services.

**Fig. 1:**
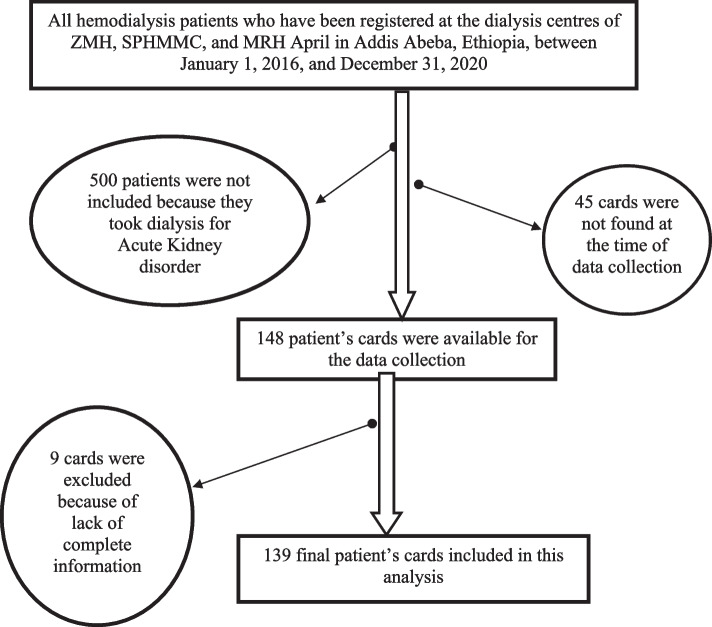
The sampling technique used to assess the survival and predictors of death among ESRD patients receiving hemodialysis in AA, Ethiopia, 2016 – 2020

All chronic kidney patients aged 18 years and above who were on hemodialysis for End-Stage Renal Disease during the study period were followed for 231 person-years. The following records were excluded: (1) Patients who starts hemodialysis for acute renal failure or patients who started dialysis on an emergency basis or acute dialysis (with the duration from first to last dialysis being less than 30 days); (2) those with incomplete medical record/charts for important variables; and (3) alive patients transferred into COVID- 19 center with their medical cards as these patients had restricted access (Fig. 1).

### Outcome of interest

The outcome was death. Patients were censored if they were lost to follow-up, immigrated, or on the 31^st^ of December 2020 when the study ended.

### Covariates

The covariates included: (1) comorbidities such as hypertension, diabetes mellitus, cancer, chronic kidney disease-mineral and bone disorder (CKD-MBD), types of vascular access for dialysis (AV Fistula, Grift, Catheter); (2) cardiovascular disease such as coronary artery disease (CAD), cardiac arrhythmia, cardiac failure, cardiac valvular disease, pericardial disease, cardiomyopathy, and congenital heart disease; (3) biochemical profile of the patients such as Serum levels of hemoglobin, creatinine, albumin, Phosphorus; (4) duration of dialysis per season and frequency of dialysis per week.

### Operational definitions

#### Chronic Kidney Disease (CKD)

Refers to GFR 60 ml/min per 1.73 m2 for 43 months Kidney damage for greater than 3 months [16].

#### Infection

Refers to bloodstream infections related to vascular access, or any hospital acquired infection like phenomena or other viral infection like hepatitis B virus.

#### Hypertension

Refers to patients is diagnosed when pre-dialysis Blood Pressure (BP) > 140/90 mmHg or when post-dialysis BP is > 130/80 mmHg [17].

#### Cardiovascular disease

Disease conditions including coronary artery disease (CAD), cardiac arrhythmia, cardiac Failure, cardiac valvular disease, pericardial disease, cardiomyopathy, and congenital heart disease [18].

#### Haemoglobin

A protein found in red blood cells that distributes oxygen to your body's organs and tissues while also carrying carbon dioxide away from them back to your lungs.

#### Albumin

The protein that the body needs to support tissue growth and repair is provided by albumin. Additionally, it can aid in fluid evacuation during the dialysis procedure. Fluid will more easily migrate from swollen tissues into the circulation if your albumin level is good, where it can subsequently be collected by the dialyzer. The ideal range for serum (blood) albumin in dialysis patients is 4.0 g/dl or above [19].

#### Vascular access

Reaching the blood for hemodialysis requires a hemodialysis access, also known as a vascular access. Through the access, blood can be transported to the dialysis machine where it is cleaned as it travels through a unique filter known as a dialyzer. A small operation is used to create an access. There are three different ways to accomplish this: (1) Fistula, which is an access created by joining an artery and vein in your arm; (2) Graft, which is an access created by using a piece of soft tube to join an artery and vein in your arm; and (3) Catheter, which is a soft tube inserted into a large vein, usually in your neck [20].

### Data source

Data were extracted from medical records and hemodialysis registration books using a pretested data extraction form. The investigators further checked for the validation and completeness of the extracted data. The variables included in this study were recorded in an Excel file. Missing values for important variables were completed by reviewing clinical notes and reports available in the selected dialysis centers. Two trained Master of Public Health students and two Bachelor of nursing from each hospital, and one supervisor were involved in the data extraction and validation. Both supervisor and data collectors were trained for two days on how to extract data, what to be extracted and to make them internalize the context of each question in the data extraction form.

### Statistical analysis

Data were entered into EpiData and exported to SPSS for further analysis. Mean or median and standard deviation were used to present numeric data and frequencies and percentages to present a categorical data.

After data management, person-time of follow-up from the date of starting hemodialysis to death, loss to follow-up, or the end of the study were calculated. The Incidence rate of death was calculated by dividing the number of deaths among CKD patients occurring by person-years of follow-up (24/231 = 104 per 1000 person-years). The actuarial life table was used to estimate survival after initiation of hemodialysis; and the Kaplan–Meier test to estimate the probability of death and the median time to death after initiation of hemodialysis. Log-rank test was employed to compare time to death between different covariates. The proportional hazard assumption was checked by using the Log (-log (St)) plots and tests. Finally, Cox proportional hazard model was fitted to determine the probability of death after initiation of hemodialysis adjusting for confounding factors. Covariates that violate the assumption of Cox proportional hazard model were excluded from the analysis. All statistically significant variables having a *p*-value ≤ 0.25 in the bivariate analysis were adjusted in the final model [21]. The crude and adjusted hazard ratio (HR) and its 95% confidence interval (CI) were estimated to identify and report significant predictors of death at a *p*-value < 0.05.

## Results

A total of 139 chronic dialysis patients who started dialysis between January 1, 2016, and December 30, 2020, were included in this study (Table 1).

**Table 1:** Sociodemographic characteristics of 139 ESRD patients on maintenance haemodialysis in Addis Ababa Ethiopia, from January 1st 2016 to December 31st 2020

Characteristic		Frequency(n)	Percentage
Age (mean ± SD (35.81 ± 11.9)	18–34	77	55.4
	35–54	48	34.5
	≥ 55	14	10.1
Gender	Male	88	63.3
	Female	51	36.7
Occupatio	Government	25	18.0
	Private	30	21.6
	Unemployed	57	41.0
	Farmer	1	0.7
	Student	6	4.3
	Not recorded	20	14.4
Residence	Addis Ababa	102	73.4
	Out of Addis Ababa	37	26.6
Marital status	Single	59	42.4
	Married	75	54.0
	Divorced	3	2.2
	Widow	2	1.4

### Socio demographic characteristic of the patients

Table 1 presents the characteristics of patients included in this study. Slightly above half of the patients were males, 88 (63.3%). The mean (± SD) age at dialysis initiation was 36.81(± 11.5) years, and more than half were in the younger age category (18 – 34 years). The majority of the patients 102(73.4%) were living in Addis Ababa while the rest 37 (26.6%) came to Addis for treatment from different regions of Ethiopia (Table 1).

### Baseline characteristics of the patients

The mean (± SD) of systolic blood pressure was 143.25 mmHg (± 22.1) at the initiation of dialysis. The mean blood pressure at the last session of dialysis was 142.10 (± 19.1 mmHg). The mean (± SD) weight of the patients at dialysis initiation of dialysis was 55.5 kg (Table 2).

**Table 2:** Base line data blood pressure and weight with their statistical parameter ESRD patients on maintenance haemodialysis for in Addis Ababa Ethiopia, from January 1st 2016 to December 31st 2020

Base line data of the patient	Mean	Median	Standard division	Range	Minimum	Maximum
Systolic BP(first)	143.95	140	21.935	129	92	221
Diastolic BP (first)	87	90	14.941	73	53	126
Systolic BP(last)	142.1	140	19	99	95	194
Diastolic BP(last)	84	86	13	86	44	130
Weight (first)	55.54	54	11.42	56.1	31.8	87.9
Weight(last)	54.9	53	10.99	57	30	87

### Treatment modality

The majority of patients (76.2%) had 4 h of dialysis three times each week. Arteriovenous fistula (AVF) accounts for 63.3% of hemodialysis patients' treatment methods, while catheter (AVC) accounts for 36%.

Twenty-four (17.3%) of the patients received a blood transfusion while receiving dialysis, and approximately 89.2% of the patients received erythropoietin (EPO). The leading causes of ESRD were hypertension (48.2%), diabetes mellitus (6.5%), glomerulonephritis (6.5%), renal stones (2.2%), and unexplained reasons accounted for 36.7% of ESRD cases (Table 3 and Table 4).

**Table 3:** Treatment modalities for 139 ESRD patients on maintenance haemodialysis in Addis Ababa Ethiopia, from January 1st 2016 to December 31st 2020

Variables	Characteristic	Frequency	Percentage
Frequency of dialysis session/week	Twice	15	10.8
	Three	124	89.2
Duration of dialysis per session	Three hours	5	3.6
	Three and half hours	13	9.4
	Four hours	121	87
Types of vascular access	Catheter	50	36
	Arteriovenous fistula	88	63.3
	Arteriovenous graft	1	0.7
Cause of CKD	Hypertension	67	48.2
	Diabetes mellitus	9	6.5
	Glomerulonephritis	9	6.5
	Renal stone	3	2.2
	Unknown	51	36.6
Blood transfusion	Yes	24	17.3
	No	115	82.7
EPO treatment	Yes	124	89.2
	No	15	10.8
Cause of death (*n* = 24)	Cardiovascular disease	5	3.6
	Infection	5	3.6
	Uremic complication	1	0.7
	Sudden death	6	4.3
	Hyperkalemia	1	0.7
	Cardiac arrest	2	1.4
	Others	4	2.8

**Table 4:** Patient status for 139 ESRD patients on maintenance haemodialysis in Addis Ababa Ethiopia, from January 1st 2016 to December 31st 2020

Variable	Category	Total (%)	Number of event (%)	Censored (%)
Sex	Male	88(63.3)	15(17)	73(83)
	Female	51(36.7)	9(17.6)	42(82.4)
Resident	Addis Ababa	102(73.4)	19(18.63)	83(81.37)
	Out of Addis	37(26.6)	5(13.51)	32(86.49)
Marital status	Married	75(54.7)	12(16)	63(84)
	Unmarried	63(43.3)	12((19)	51(81)
Vascular access	AVF	88(63.3)	6(6.8)	82(93.2)
	Catheter	50(36)	17(34)	33(66)
	Graft	1(0.7)	1(100)	0(0)
Frequency of dialysis per week	Twice per week	15(10.8)	7(46.7)	8(53.3)
	Three per week	124(89.2)	17(13.7)	107(86.3)
Duration of dialysis	Three hours	5(3.6)	2(40)	3(60)
	Three and half hour	13(9.4)	4(30.8)	9(69.2)
	Four hours	121(87)	18((14.9)	10,385.1)
Hypertension	Yes	93(66.9)	19(23)	74(77)
	No	46(33.1)	5(4.65)	41(95.35)
Diabetes mellitus	Yes	11(7.9)	3(27.3)	8(72.7)
	No	128(92.1)	21(16.4)	107(83.6)
albumin(mg/dl)	Under 3.5	57(41)	18(31.6)	39(68.4)
	3.51–4.00	45(32.4)	2(4.4)	43(95.6)
	Above 4.01	37(26.6)	4(10.8)	33(89.2)

### Comorbidity and complication

The most frequent comorbidity was hypertension, which occurred in 93 cases (66.9%), followed by cardiovascular disease, 74 cases (53.2%), diabetes mellitus, 11 cases (7.9%), and strokes, 3 cases (2.2%). Seventy-eight (56.1%) of the patients were developed different types of infections sometimes in their course of treatment. For example, Hypotension occurred in (36.0%) of the patients at least once during the period of dialysis (Table 3).

### Survival status of patients on haemodialysis

One hundred thirty-nine (139) patients were followed for a total of 2772 person-months during the course of the study. Of these patients, 88 (63.3%) were male and the mean age (± SD) of the patients was 36.8 (± 11.9) years. At the end of the study, 24 (17%) of the patients died, 67 (48.2%) were alive, 43 (30.9%) performed transplants, and 5 (3.6%) defaulted. The mean survival time was 46.2 months (95% CI: 41.8–50.5). The incidence of Mortality at the end of follow-up is estimated at 104 per 1000 person-years. The likelihood that these patients would survive for one and 5 years was 91% and 65%, respectively.

### Predictors of mortality

**Fig. 2:**
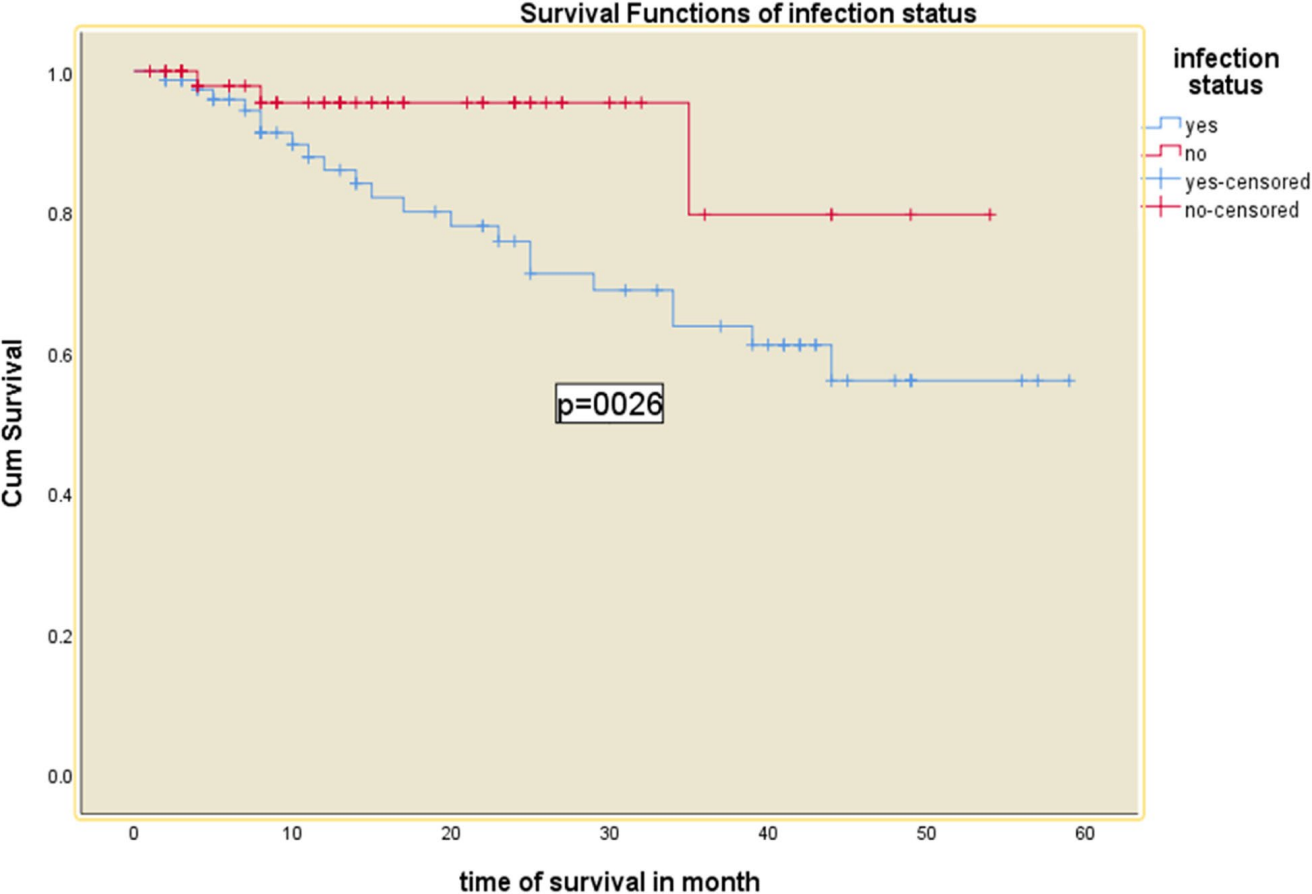
Kaplan–Meier survival curves for hemodialysis patients with respect to the covariate infection status in Addis Ababa, Ethiopia from 2016–2020

**Fig. 3:**
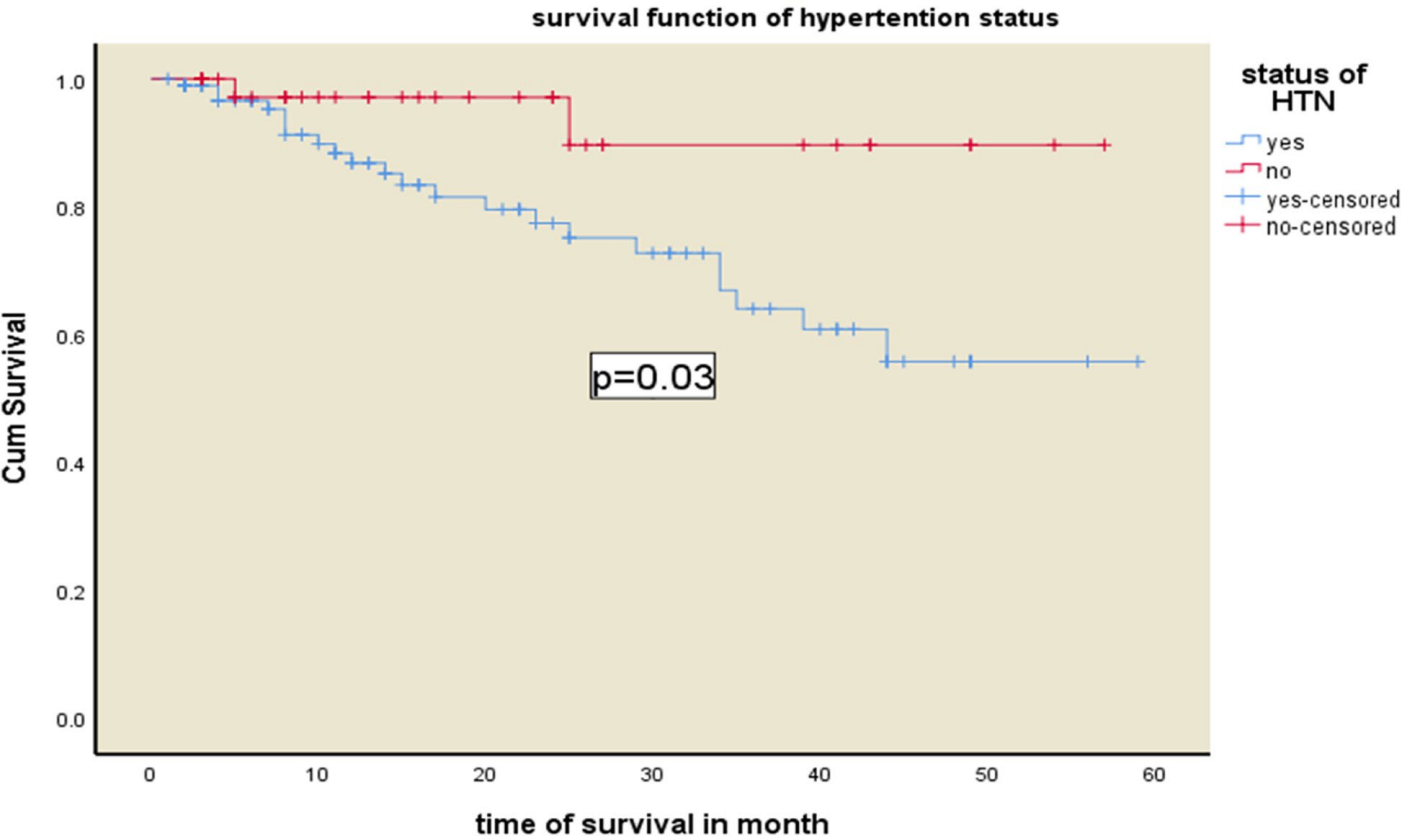
Kaplan–Meier survival curves for hemodialysis patients with respect to the covariate status of hypertension in Addis Ababa, Ethiopia from 2016–2020

**Fig. 4:**
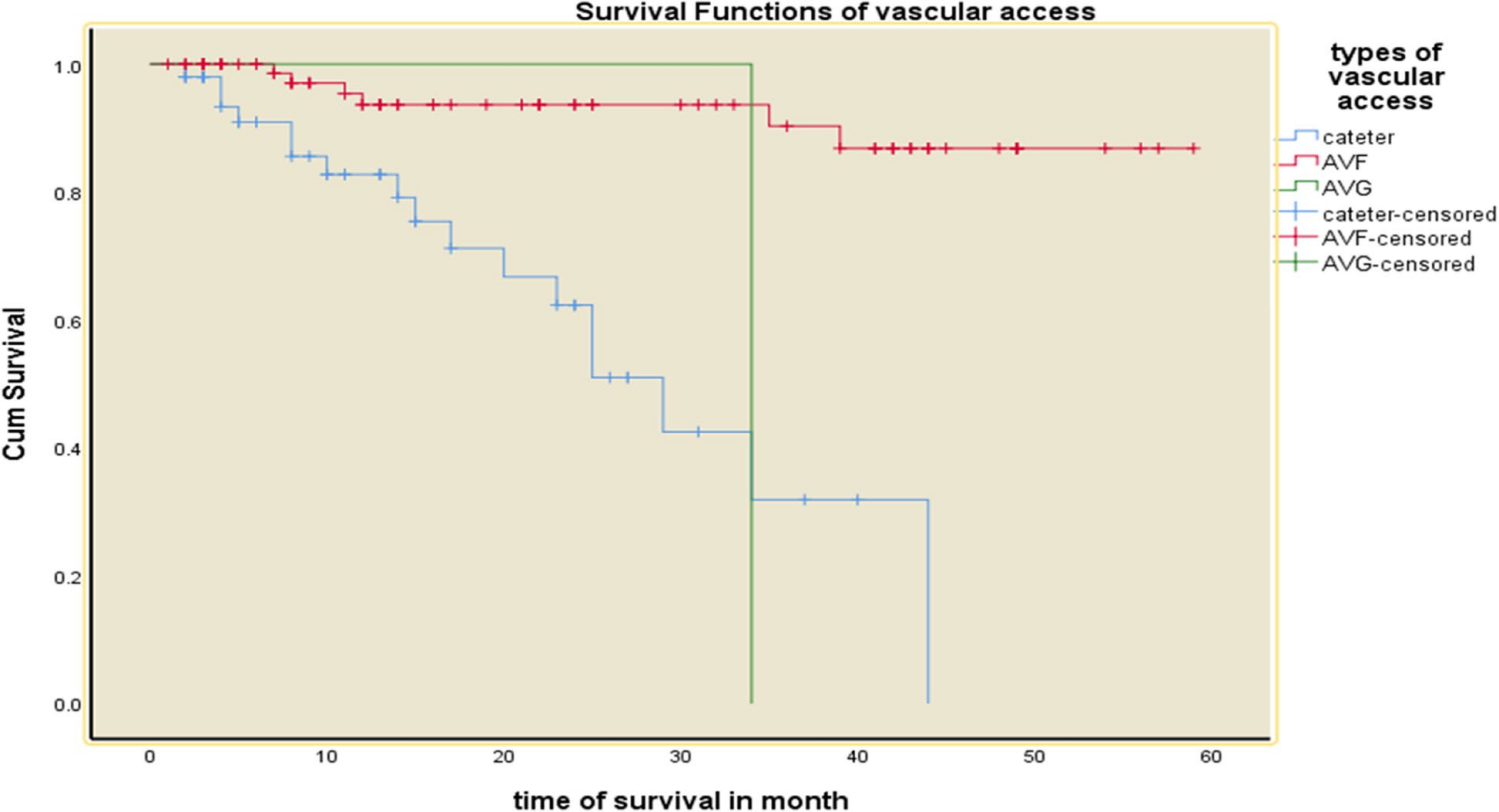
Kaplan–Meier survival curves for hemodialysis patients with respect to the covariate types of vascular access in Addis Ababa, Ethiopia from 2016–2020

**Fig.5:**
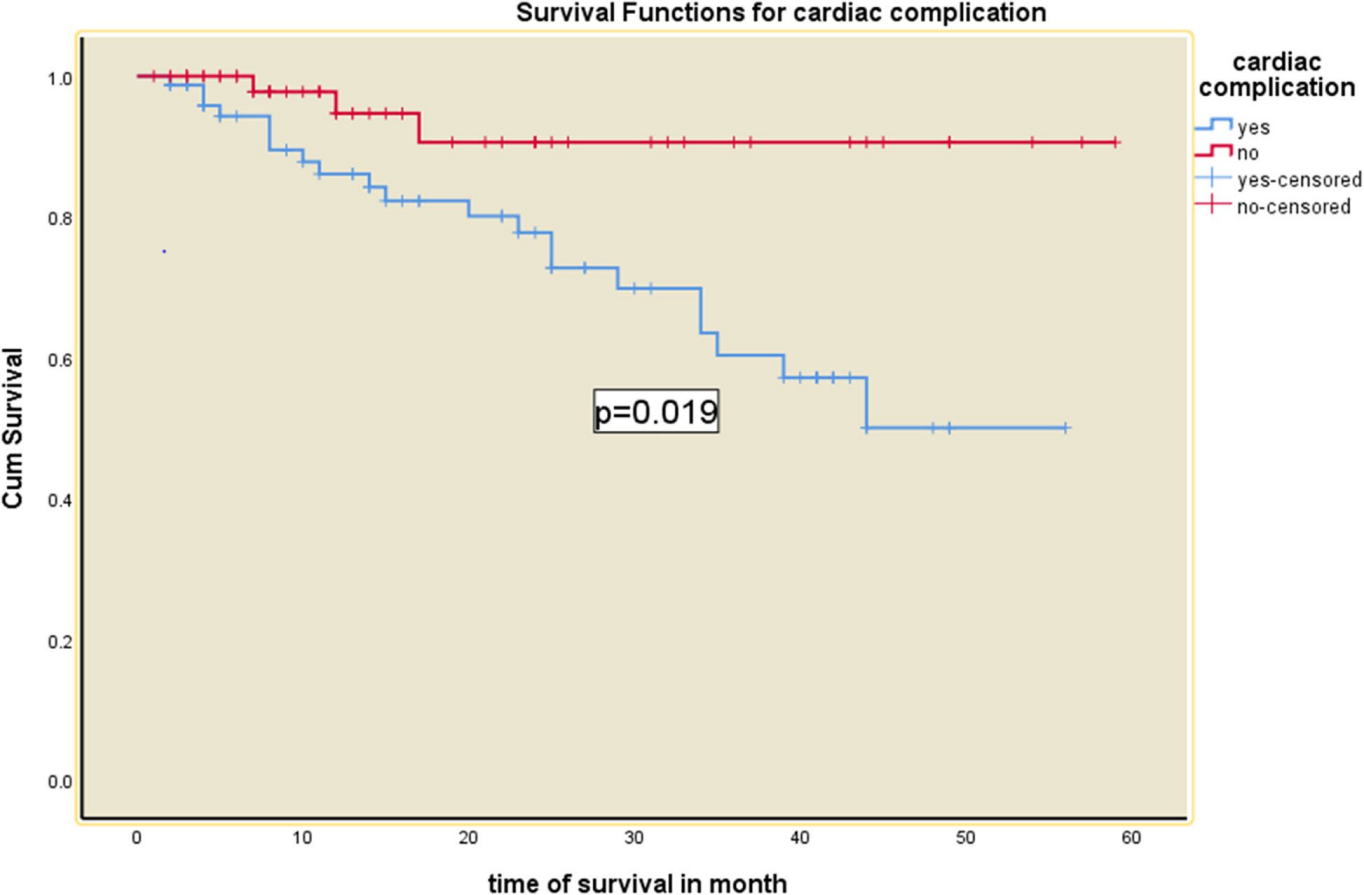
Kaplan–Meier survival curves for hemodialysis patients with respect to the covariate cardiac complication in Addis Ababa, Ethiopia from 2016–2020

**Fig. 6:**
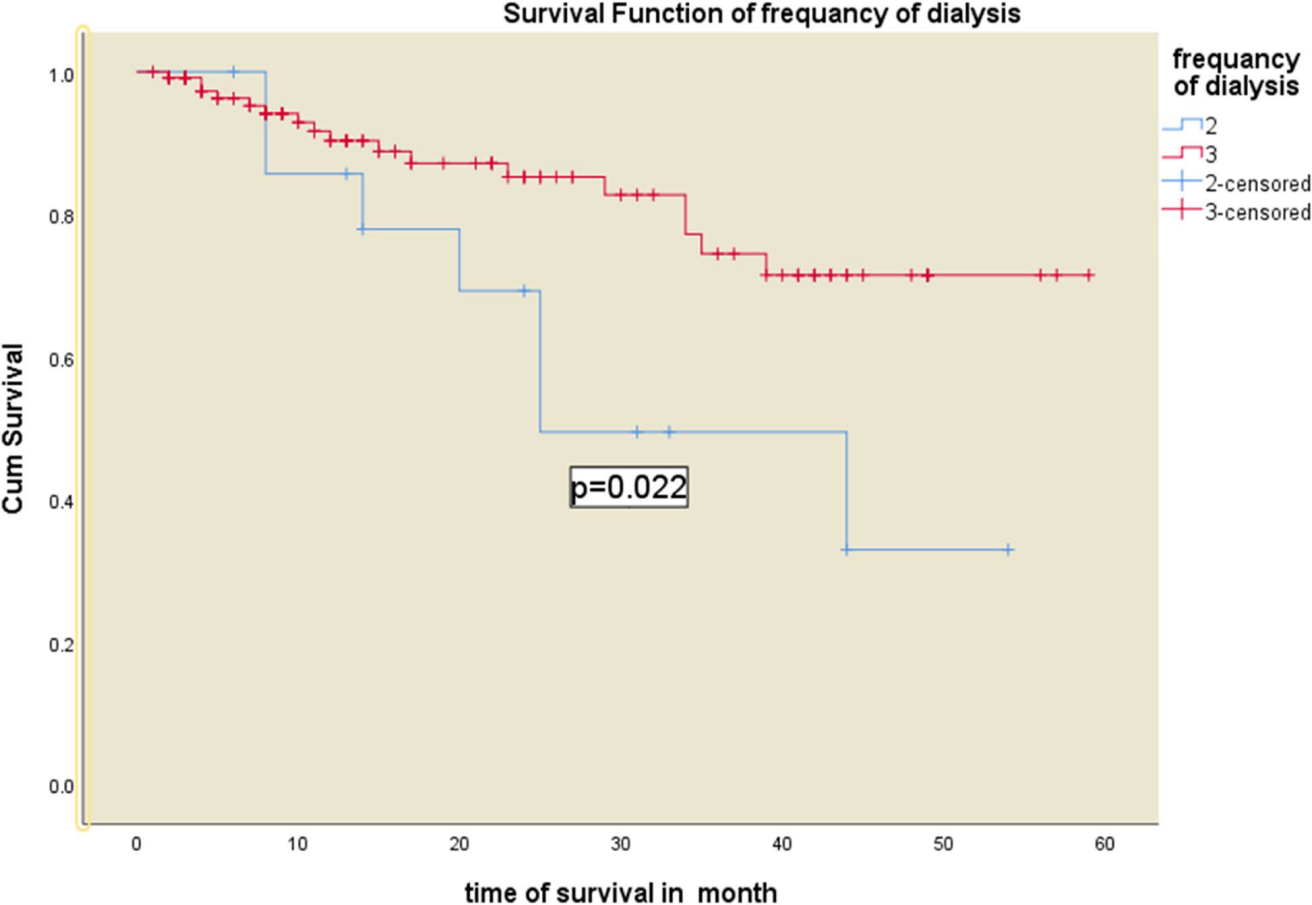
Kaplan–Meier survival curves for hemodialysis patients with respect to the covariate frequency of dialysis per week in Addis Ababa, Ethiopia from 2016–2020

Hypertension, cardiovascular complication, infection, and types of vascular access were turned-out to predict the mortality in the adjusted model (Figs. 2, 3, 4, 5 and 6 and Table 5). Being hypertensive increased the risk of mortality by 4.3 times (95% CI: 1.02—18.43).

**Table 5:** Cox Proportional Hazard Model of ESRD patients on maintenance hemodialysis in Addis Ababa Ethiopia, from January 1st 2016 to December 31st 2020

Covariates	β	SE	Wald	Sig	Exp.(β)/ AHR	95% CI for Exp.(β)	
Hypertension							
Yes	1.966	0.805	5.965	0.015*	7.139	1.474	34.565
No (Ref)							
Types of vascular access							
Graft	2.974	1.162	6.553	0.01*	19.568	2.008	190.736
Catheter	1.345	0.513	6.868	0.09*	3.839	1.404	10.497
Fistula (Ref)							
Infection							
Yes	1.277	0.659	3.752	0.035*	3.586	1.985	13.054
No (Ref)							
Cardiovascular complication							
Yes	1.547	0.490	5.676	0.017*	4.696	1.316	16.763
No (Ref)							

*AHR* Adjusted Hazard Ratio, and Ref is the reference group, *β* Estimated Regression Coefficient, *SE* Standard error of β, *CI* Confidence Interval, *Sig*. Statistical Significance, and *Exp. (β)* Exponent of Beta = Hazard Ratio

^*^Significant (*P*-value < 0.05)

Patients with cardiovascular diseases had a 4.7 (95% CI: 1.32—16.76) times higher risk of mortality. The risk of mortality among patients using Arterio-Venous Graft and the temporary venous catheter was 19.57 (95%CI: 2.01–190.74) and 3.84 (95% CI: 1.40, 10.50) times higher than those patients using Arterio-Venous Fistula. Furthermore, the risk of mortality was 3.59 (95% CI: 1.98–13.05) times at higher risk among patients with comorbid infection (Table 5).

## Discussion

According to this analysis, a total of 139 chronic hemodialysis patients began receiving treatment between January 1, 2016, and December 31, 2020. Out of these, 110 (79.1%) were still alive, 24 (17.3%) were deceased, and 5 (3.6%) were in default. The median survival duration was 46.2 (95% CI: 41.8, 50.5) months. According to this study, the type of vascular access, the patient's hypertension status, the patient's infection status, and cardiovascular diseases were found to be predictive of death among hemodialysis patients.

The patients' median follow-up time was 46.2 months, which is consistent with the 43-month estimate for patients at Adama Hospital [22]. Furthermore, the survival percentage of patients under follow-up was 70% at five years and 86 percent at one year, which is comparable to an 80 percent survival rate reported in a similar study elsewhere [23]. Another Brazilian study found an 84.7 percent first-year survival rate and a 63.3 percent five-year survival rate [24]. The main cause of the disparity could be Ethiopia's poor service accessibility and pricing. The patients' reduced long-term survival rate could be ascribed to the fact that they are on dialysis, which is very low and can be attributed to medical factors such as vascular access type, hypertension, infection status, and cardiac issues, which we have observed.

When compared to patients who did not have cardiovascular issues, patients with cardiovascular diseases had a fourfold greater risk of death. In another way, patients with a heart problem had a 14-month shorter average survival time. This finding is comparable to that of a Chinese study, in which cardiovascular diseases were found to be a statistically significant predictor of mortality in ESRD patients [25]. Similarly, we discovered a three-fold increased risk of mortality among patients with cardiovascular diseases. It has been observed that stroke was a significant predictor of all-cause mortality in patients with cardiovascular problems [25]. The impact of chronic renal disease on mortality is substantial and growing, necessitating optimum medical care. According to studies, people with renal insufficiency have a higher rate of cardiovascular morbidity and mortality [26].

Infected ESRD patients had a three-fold increased risk of death compared to hemodialysis patients who were not infected. In a similar study in Ethiopia, patients on maintenance hemodialysis with septicemia had a higher risk of death, accounting for 34.1 percent of all-cause mortality [7]. Another Taiwanese study discovered a risk that is lesser but in the same direction. Infection combined with severe CKD raised the chance of death from all causes by 34% in the first year and 19% over the nine-year follow-up period [27]. Efforts to reduce infectious complications and prevent infection at any level should improve hemodialysis patient survival.

Another feature that was revealed as a predictor of death among hemodialysis patients in the current investigation was the kind of vascular access used for hemodialysis treatment. Patients with a Central Venous Catheter had a fourfold increased risk of death compared to those with AFV access. This finding is in line with research in Iran that found a 3.6-fold increased risk of death [28]. This study found that vascular access via catheter was associated with a higher risk of death, which is most likely due to the fact that. In fact, reducing the use of catheters helped patients avoid infection issues. Another study found that using a catheter was linked to a 67 percent higher risk of death when compared to using an AVF [26]. Similarly, we discovered that patients who utilized Catheter had a 47 percent increased chance of death.

In contrast to our findings, a retrospective cohort research in Belgium indicated that the type of vascular access was not independently linked with patient survival, even after accounting for changes in vascular access over time [29]. In addition, the results of this study are consistent with a nighty one hemodialysis case summary of hemodialysis patients in Ethiopia, which revealed that patients who had catheter as permanent vascular access had a poor outcome when compared to artery venous fistula and Graft, with one-year survival rate of 5.4 percent for AVC group and 66.9% for other vascular access types (*p*-value < 0.05) [7]. In conclusion, most investigations have discovered that using an arteriovenous catheter increases the risk of death when compared to other vascular access methods. This could be owing to the high infection risk in the area.

Hypertension was another important predictor of death, with a fourfold increase in the likelihood of dying. In our study, high blood pressure was linked to a fourfold increased risk of death, which contradicts research that indicated the opposite relationship, that is, high blood pressure was linked to a low risk of death, and low blood pressure was linked to a high risk of death in dialysis patients. In contrast to this study, some previous research refers to this phenomenon as "reverse epidemiology," implying a paradoxical link between mortality and the effect of hypertension in dialysis patients. The paradoxical phenomena of lower blood pressure or a reduction in blood pressure over time being related to increased mortality, while higher blood pressure is associated with lower mortality, is referred to as "reverse" hypertension epidemiology [18].

In the literature, there are various plausible explanations for this finding, including discrete (binary) categorization of hypertension at the baseline as (yes or no) rather than using blood pressure as a continuous variable (SBP, DBP). The severity of hypertension was not revealed by categorizing it into two categories at the start. Due to a lack of information on blood pressure measures and the duration of antihypertensive drug use or any other relevant treatment at the baseline, normotensive or well-controlled hypertension patients could have been classified as hypertensive. In a research conducted in Indonesia, high blood pressure was linked to a significant risk of mortality, however, the risk was lower than in our study [24].

Overall, this study can provide early information on variables that affect the survival status of CKD patients receiving hemodialysis in Addis Ababa, one of Ethiopia's three hemodialysis centers. Because we know so little about this subject, our research will be critical in identifying difficulties and complications in hemodialysis patients. However, because of the study's small sample size, it can't be sure how reliable this Information is scarce, so further research to overcome the study's apparent limitations should be implemented. Because this was a retrospective analysis, it is difficult to identify certain crucial factors that should have been taken into account, raising the possibility of residual confounding bias.

### Limitations of the study

Because this is a retrospective study using secondary data, certain crucial factors cannot be obtained, which limits the generality of these findings. For example, medication and treatment history for the specific comorbidity disease cannot be recorded in people with comorbidity disease, which may reduce the study's power. The study's weakness is that it only looks at the overall survival of hemodialysis participants. This could skew our findings by overestimating the death rate. One explanation is that some patients' deaths can be caused by factors other than renal disease or hemodialysis, such as trauma or acute sickness. As a result, it will be critical to assess chronic renal disease-specific mortality, which will aid in determining the actual survival probability of these patients when other causes of death are ruled out.

Furthermore, the cause for patients who were lost to follow-up cannot be ascertained, which would provide us with helpful information if it were collected. Because they are admitted for treatment and all documents are transferred to the COVID-19 center, some patients are excluded from the study due to concerns about COVID-19 cross-contamination, and because the document is restricted for data collection due to fear of exposure could result in important variables are missed. Moreover, certain confidence intervals were quite large. This could be owing to a smaller sample size or a lesser number of frequencies in the cell, both of which reduce the study's power. As a result, we'd like to identify these as a study's intrinsic weaknesses. Despite the fact that this study has intrinsic limitations, those restrictions have no bearing on the generalizability of the findings up to this point.

## Conclusions

The hemodialysis patients' death rate in the chosen dialysis facilities was high. Preventing and treating comorbidities and complications during dialysis would probably reduce the mortality of CKD patients. Furthermore, the best way to avoid and manage chronic kidney disease is to take a complete and integrated approach to manage hypertension, diabetes, and obesity.

## Data Availability

The datasets used and/or analyzed during the current study available from the corresponding author on reasonable request.
